# Fish Responses to Total Dissolved Gas Supersaturation: From the Perspective of Historical Experimental Data in the Upper Yangtze River

**DOI:** 10.3390/ani16213423

**Published:** 2026-10-29

**Authors:** Jianing Rao, Rongxuan Pan, Yuanming Wang, Jingjie Feng, Xintong Li, Kefeng Li

**Affiliations:** 1 State Key Laboratory of Hydraulics and Mountain River Engineering, Sichuan University, Chengdu 610065, China; 2 Sichuan Academy of Water Conservancy, Chengdu 610072, China

**Keywords:** total dissolved gas supersaturation, fish tolerance, fish avoidance, predictive formula, upper Yangtze River

## Abstract

During discharge from high dams, the high-velocity water flows mix with air, causing downstream total dissolved gas (TDG) supersaturation, threatening fish survival. However, a systematic understanding on fish response patterns under the coupled influence of multiple environmental and biological factors remains limited. This study synthesized historical experimental data to explore fish tolerance and avoidance in response to TDG supersaturation in the upper Yangtze River. Economic fish species demonstrated overall stronger tolerance than the rare fish species under all TDG levels. Significant interspecific differences in economic fish tolerance were observed with increasing TDG levels. *Aristichthys nobilis* demonstrated stronger tolerance than *Hypophthalmichthys molitrix*. For the rare fish, *Procypris rabaudi* showed the weakest tolerance. Exposure time, TDG level, species and water temperature were identified as the primary factors influencing fish mortality. Based on these, mortality prediction formulas for rare and economic fish were established, achieving an *R*^2^ over 0.59. The avoidance ability of fish increased significantly with increasing TDG levels and reached 50% at 130% TDG. Significant interspecific differences in fish avoidance were observed at a 140% TDG level or above, and *Schizothorax prenanti* showed the weakest avoidance ability. TDG level was the dominant factor influencing fish avoidance ability, followed by body length. Accordingly, a prediction formula for fish avoidance rates was proposed with an *R*^2^ of 0.88. The findings offer critical insights for ecological risk management and the development of fish conservation strategies downstream of high dams.

## Simple Summary

During discharge from high dams, the high-velocity water flows mix with air, causing downstream total dissolved gas supersaturation, threatening fish survival. In response to the issue of gas supersaturation caused by intensive hydropower development in the upper Yangtze River, numerous laboratory experiments and field observations have been conducted with a focus on the tolerance and avoidance patterns of endemic fish. To better understand how fish respond to this stress, we analyzed a large set of historical experimental data. We found that commercial fish species generally tolerate supersaturated water better than rare fish. Among all species tested, *Aristichthys nobilis* showed the strongest tolerance, while a rare species called *Procypris rabaudi* was the most sensitive. As the gas levels increased, fish became more likely to swim away and exhibited avoidance towards gas supersaturation. Most species started avoiding the water when gas levels reached 130%. We also developed simple formulas to predict how likely a fish is to die or escape under different conditions. These findings can help dam operators manage water releases more safely and design better fish refuges downstream, ultimately supporting the protection of fish biodiversity in rivers affected by hydropower development.

## 1. Introduction

Total dissolved gases (TDGs) are supersaturated when the pressures of gases in a solution exceed ambient barometric pressure, with TDG saturation conventionally calculated as the ratio of the pressures of gases in a solution to the pressures in the atmosphere [1]. During dam discharges, large amounts of air are entrained and dissolved in the downstream deep-plunge pool under the high pressure. These dissolved gases can not be effectively released upon outflow from the deep-plunge pool to the downstream river, thereby leading to TDG supersaturation [2]. The supersaturated TDG enters fish through the gills, triggering vascular blockages and tissue damage, which results in gas bubble trauma and ultimately fish mortality [3]. Supersaturated nitrogen, which is not well absorbed or metabolized by fish, is generally recognized as the primary cause of gas bubble trauma, while oxygen or carbon dioxide supersaturation caused by dam discharge does not have a lethal effect on fish [3,4,5,6]. The impact of TDG supersaturation on fish has attracted widespread attention. Incidents of fish mortality induced by TDG supersaturation have been documented in the Columbia River basin in North America, the Otra River basin in Europe, the Amazon River basin in South America, and the Yangtze River basin in China. TDG supersaturation generated by high dams has become a significant survival risk for fish in dam-regulated systems worldwide [7,8,9].

Fish tolerance to TDG supersaturation is influenced by multiple environmental and biological factors [2]. TDG level plays a critical role in determining survival. In the Columbia River basin, rainbow trout, sockeye salmon, and white sturgeon exhibited mortality of 10–20% under 120% TDG supersaturation, whereas mortality increased to 26–61% under 130% TDG supersaturation [10]. Hydrostatic pressure rises with water depth, effectively reducing the relative TDG level and offering survival compensation for fish. Specifically, each 1 m increase in depth corresponds to an approximate 9.8% decrease in TDG level [11]. Differences in water temperature also influence fish tolerance under the same TDG level [12]. Biological characteristics, including age and body size, are also important factors affecting fish survival in TDG-supersaturated water. Juveniles demonstrated greater tolerance than adults under high TDG levels [13].

Bubbles in TDG-supersaturated water stimulate the lateral line nervous system of fish, triggering avoidance behavior [14]. This enables fish to migrate to downstream areas or tributaries with lower TDG levels, thereby escaping the stress of high TDG levels [15]. The ability to perceive and avoid TDG-supersaturated water varies considerably among fish species. *Oncorhynchus tshawytscha* can detect TDG saturation as low as 117% and effectively avoid levels of 130% to ensure survival, whereas *Oncorhynchus kisutch* lacks the ability to sense TDG supersaturation [16,17]. Detection capabilities also vary with TDG levels. *Oncorhynchus mykiss* can avoid TDG levels ranging from 125% to 145%, but fails to effectively avoid water with a TDG level of 115% [14,18].

There is considerable overlap between areas rich in hydropower and the habitats of rare and endemic fish in China, creating a contradiction between economic development and ecological conservation [19]. The intensive proliferation of hydropower constructions has exacerbated the issue of TDG supersaturation, posing a persistent threat to aquatic ecosystems, not only in river systems with constructed hydropower but also in river basins with planning hydropower [20]. Assessment of the ecological impacts of TDG supersaturation is still urgently required in these projects, while conducting repetitive laboratory or field experiments is prohibitively expensive and time-consuming. There is a pressing need for a generalizable quantitative framework that can predict fish responses under TDG supersaturation. Moreover, key influencing factors have not been systematically identified, and quantitative relationships between fish response characteristics and these factors have yet to be established. Based on historical experimental data on the tolerance and avoidance of fish in the upper Yangtze River under TDG supersaturation, this study quantified interspecific differences in tolerance and avoidance capabilities and established predictive formulas for mortality and avoidance among fish species in TDG-supersaturated water. The results of this study not only provide quantitative support for the selection and design of fish refuge areas downstream of high dams, but also offer critical support for decision-making regarding ecological risk management, thereby enhancing the effectiveness of conservation efforts.

## 2. Materials and Methods

### 2.1. Data Collection

To explore the impacts of TDG supersaturation on endemic fish species in the upper Yangtze River, we compiled a TDG tolerance database and a TDG avoidance database on juveniles from well-controlled experiments between 2010 and 2024 [12,20,21,22,23,24,25,26,27], all of which focused on endemic fish in the upper reaches of the Yangtze River. The TDG tolerance database had 2440 fish’s survival time under TDG supersaturation and mortality under exposure time. The TDG avoidance database covered 220 avoidance rates of fish to TDG supersaturation, and each avoidance rate was tested with 20 fish. The experimental conditions such as TDG level, water depth and water temperature were also included into these two databases (see the details in the Supplementary Materials). The TDG levels set in previous experiments, which ranged from 120% to 150%, were well controlled to match the TDG levels observed during real flood discharge events in the field. The water depth in previous tolerance tests was maintained at 20–35 cm, which is relatively shallow compared to natural river depths. The water temperature in previous experiments ranged from 12 to 25 °C (Table 1). Previous laboratory experiments rarely described the flow velocity of experimental water, but they usually confined fish within nearly stationary water to test their tolerance and put fish into partially separated tanks with low flow velocity to test their avoidance.

Five fish species including *Procypris rabaudi* (*P.R.*), *Schizothorax prenanti* (*S.P.*), *Myxocyprinus asiaticus* (*M.A.*), *Hypophthalmichthys molitrix* (*H.M.*) and *Aristichthys nobilis* (*A.N.*), were used to test fish tolerance to TDG supersaturation. Among them, *P.R.*, *S.P*. and *M.A.* are the rare fish species distributed throughout the upper Yangtze River. Specially, *M.A.* is the only species of *Catostomidae* found in China, and the species was recently listed in the *China Red Data Book of Endangered Animals* [28]. The other two species, *Hypophthalmichthys molitrix* (*H.M.*) and *Aristichthys nobilis* (*A.N.*), are the main commercial fish species in the upper Yangtze River, active in the upper layer of the river. In addition, three rare fish species (*S.P.*, *M.A.*, and *P.R.*) were used to conduct fish avoidance to TDG supersaturation in previous experiments.

### 2.2. Identification of Key Factors and Construction of Mathematical Formulas

The Kaplan–Meier method, which accurately incorporates both complete and censored data, allowing for dynamic estimation of cumulative mortality over exposure time, was used to analyze the mortality of different fish under the same TDG conditions. Pairwise comparisons in survival curves between species were performed using the log-rank test, where *p*-values and χ^2^ were adjusted using the Benjamini–Hochberg false discovery rate (FDR) correction to control for Type I error. The effect of TDG exposure on the fish avoidance was tested by one-way ANOVA, Kruskal–Wallis test and the independent samples *t*-test, depending on the outcomes of normality and homogeneity of variance tests. For data meeting all parametric assumptions (Shapiro–Wilk test, *p* > 0.05; Levene’s test, *p* > 0.05), a one-way ANOVA with LSD post hoc testing or the independent samples *t*-test was utilized. Otherwise, the non-parametric Kruskal–Wallis test coupled with Tamhane’s T2 for post hoc analysis was employed. This part of the data analysis was completed in SPSS 26 and the significant difference level was set at *p* < 0.05.

Survival time and mortality were treated as the characteristic matrix for fish tolerance in TDG-supersaturated water, and redundancy analysis (RDA) indicated by the *p* value and Mantel’s test indicated by Mantel’s *r* and *p* values were employed to identify factors affecting fish tolerance in TDG-supersaturated water. The potential factors include exposure time, TDG level, water temperature, water depth, fish length, fish weight and relative fatness. Relative fatness was derived from the available length and weight data, calculated as *K* = 100 × (*W*/*L*^3^), where *W* is body weight (g) and *L* is body length (cm). Hierarchical partitioning via linear regression and random forest, indicated by contribution value and SHAP value, respectively, were subsequently employed to evaluate the weight of each factor on the mortality of endemic fish in the upper Yangtze River. For fish avoidance, hierarchical partitioning via linear regression and random forest were also applied. This part of the data analysis was completed in R 4.2.2.

After identifying the factors significantly affecting fish mortality and horizontal avoidance rates, 1stOpt software (version 10.0, 7D-Soft High Technology Inc., Beijing, China) was then employed to fit experimental data with the Levenberg–Marquardt algorithm and a universal global optimization method. Utilizing the 2440 collected sets of rare and economic fish tolerance data and 4400 sets of rare fish avoidance data, mathematical formulas were ultimately established, which could be used to calculate the mortality and horizontal avoidance rate of fish in TDG-supersaturated water.

## 3. Results

### 3.1. The Response of Fish in the Upper Yangtze River Under TDG Supersaturation

**Figure 1:**
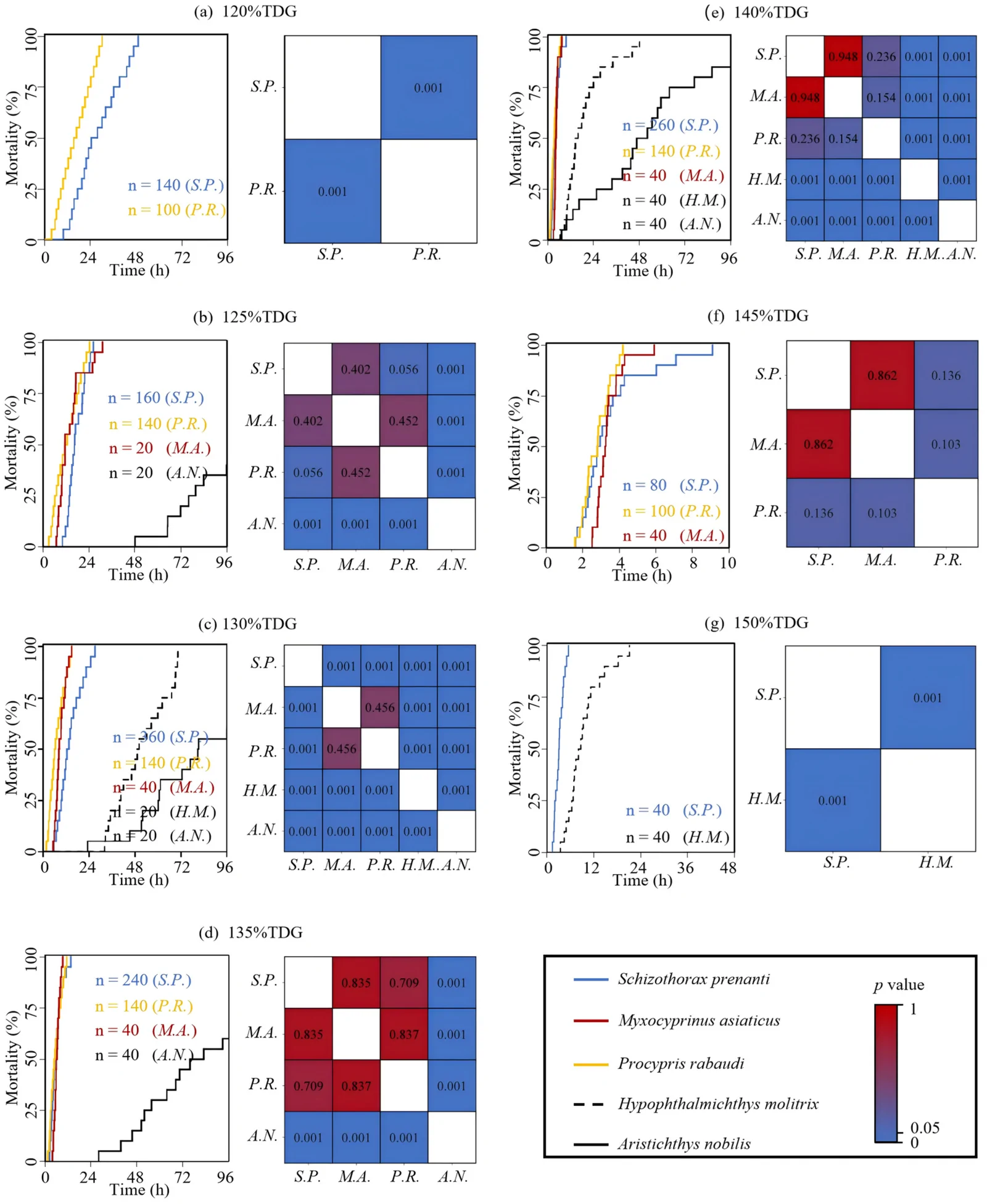
Fish tolerance in the upper Yangtze River to TDG supersaturation. (**a**–**g**) represent the interspecific differences of fish mortality in water with 120%, 125%, 130%, 135%, 140%, 145% and 150% TDG, respectively. Heatmaps show the *p*-values of pairwise comparisons in survival curves between species. The letter n with different colors indicates sample sizes of different species.

As shown in Figure 1, economic fish species (*H.M.*, *A.N.*) demonstrated overall stronger tolerance than the rare fish species (*S.P.*, *M.A.*, *P.R.*) under all TDG levels (*p* < 0.05). Among rare fish, there existed no significant difference in mortality under TDG levels from 125% to 150% (*p* > 0.05), with *S.P.* showing significantly higher tolerance than *P.R.* under the TDG level of 120% (χ^2^ = 11.494, *p* < 0.05). For two economic fish species, *A.N.* demonstrated significantly stronger tolerance than *H.M.* (*p* > 0.05). Thus, *A.N.* demonstrated the strongest tolerance, followed by *H.M.*, *S.P.*, *M.A.* and *P.R*.

**Figure 2:**
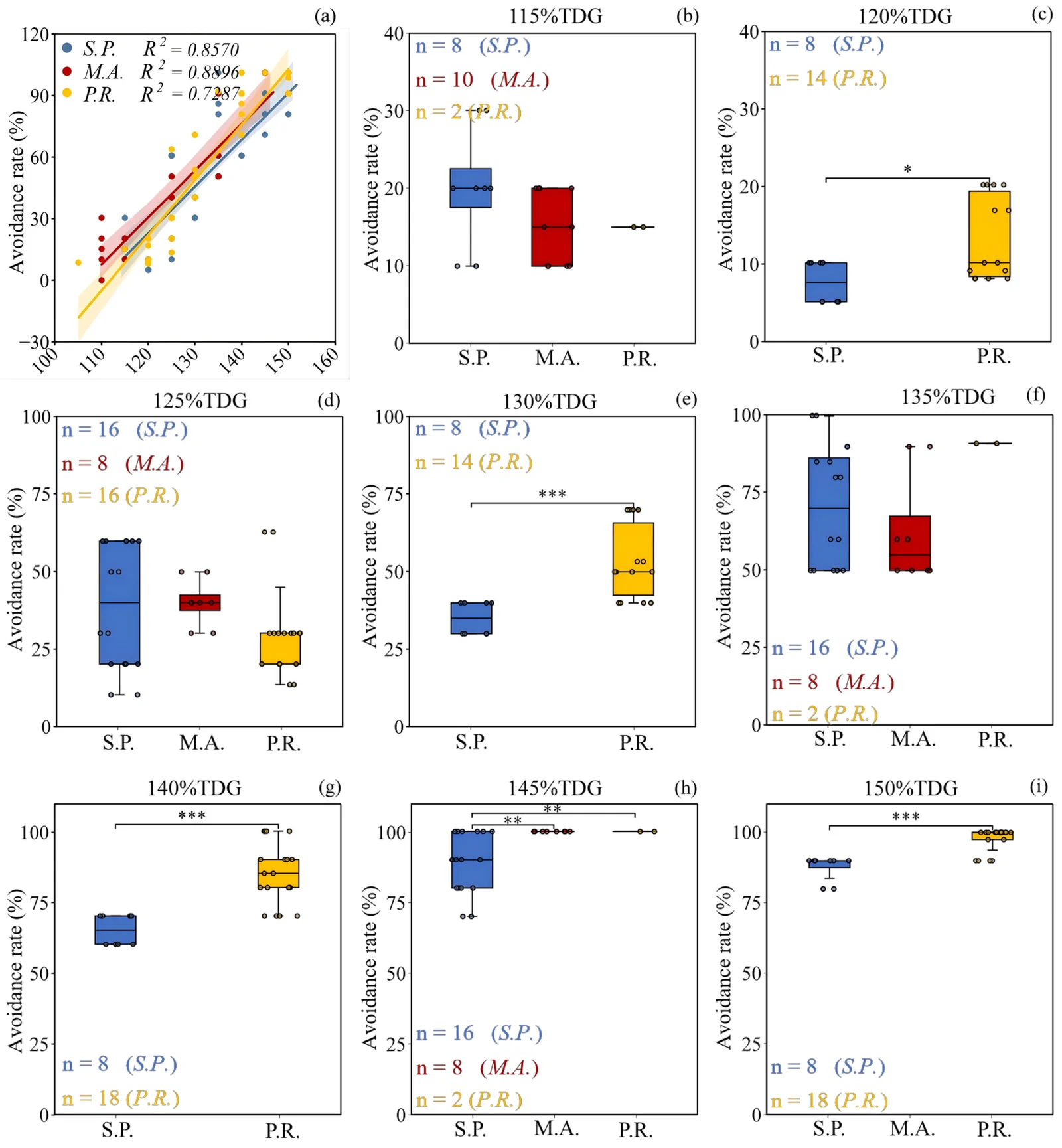
The avoidance of three rare fish species in the upper Yangtze River to TDG supersaturation. (**a**) represents scatter plots, trend lines, and confidence intervals of historical experimental data in water with different TDG levels. (**b**–**i**) represent the interspecific differences of fish avoidance in water with 115%, 120%, 125%, 130%, 135%, 140%, 145% and 150% TDG, respectively. The asterisks indicate significant differences in avoidance between two species (* indicates *p* < 0.05, ** indicates *p* < 0.01, and *** indicates *p* < 0.001). The dots and letters n in (**b**–**i**) indicate sample sizes of different species.

The avoidance abilities of *S.P.*, *P.R.*, and *M.A.* exhibited a linear increasing trend with increasing TDG levels (Figure 2). For all three rare fish species, their avoidance rate exceeded 50% at a TDG level of 130% or above. In terms of interspecific difference in fish avoidance, three rare fish species exhibited no significance when the TDG level was lower than 140%, except for *S.P.* and *P.R.* under 120% and 130% TDG. *P.R.* showed stronger avoidance ability than *S.P.* at a TDG level of 140% or above. *M.A.* also showed stronger avoidance ability than *S.P.* at a TDG level of 145%.

### 3.2. Key Factors and Mathematical Formulas for Fish Tolerance in TDG-Supersaturated Water

**Figure 3:**
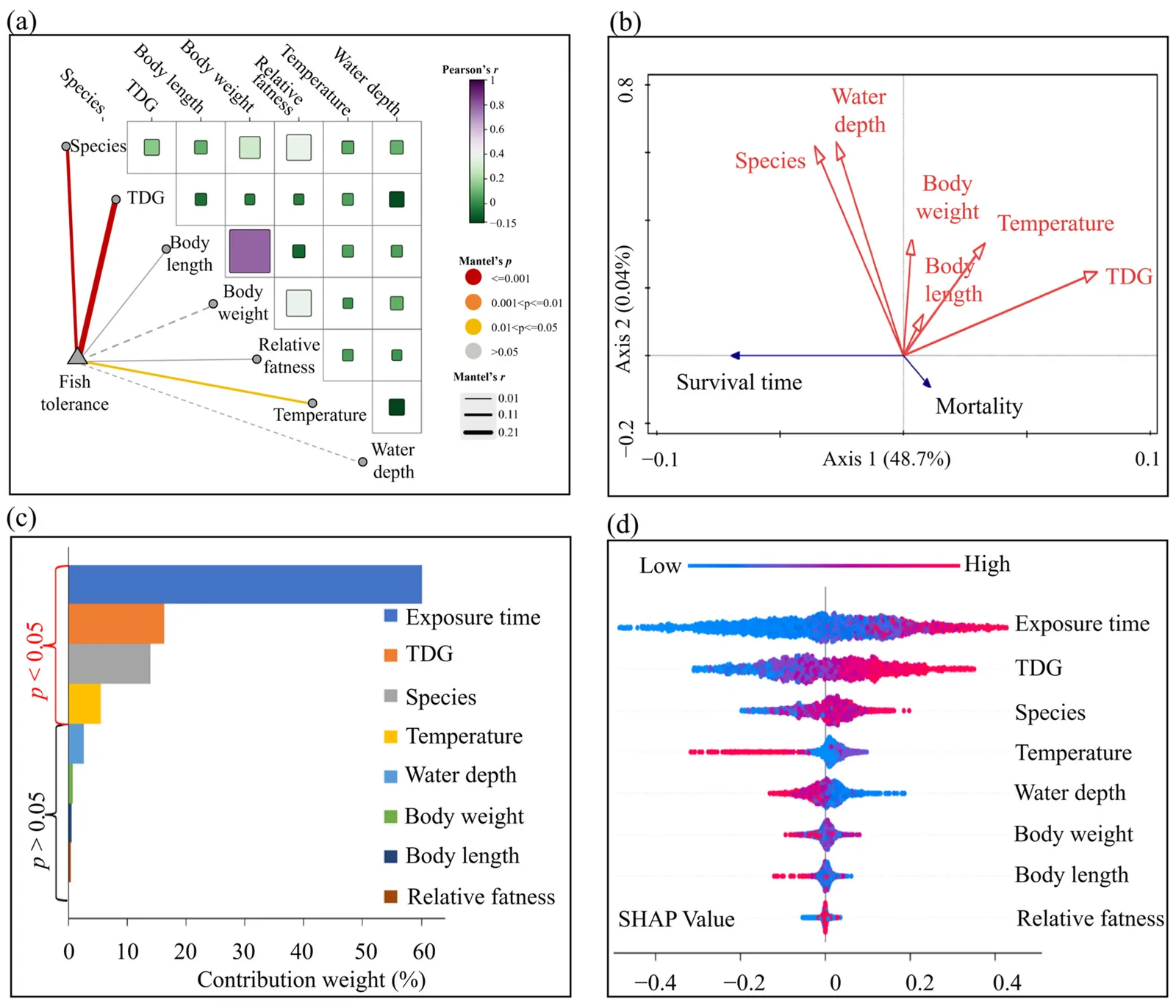
Factors affecting the tolerance of fish to TDG supersaturation. (**a**,**b**) represent the relationship between fish tolerance and factors by Mantel’s test and RDA, respectively. (**c**,**d**) indicate the factors affecting fish mortality by hierarchical partitioning via linear regression and random forest, respectively.

The result of Mantel’s test indicates that TDG level (*r* = 0.291, *p* = 0.001), species (*r* = 0.147, *p* = 0.001) and water temperature (*r* = 0.057, *p* = 0.030) significantly influenced the tolerance of fish in the upper Yangtze River to TDG supersaturation. Fish length (*r* = 0.011, *p* = 0.314), fish weight (*r* = −0.064, *p* = 0.990), relative fatness (*r* = 0.021, *p* = 0.187) and water depth (*r* = −0.010, *p* = 0.611) showed no significant correlation with fish tolerance (Figure 3a). The result of the RDA model demonstrates high reliability for the tolerance of fish in the upper Yangtze River, with the first two axes cumulatively explaining 48.74% of the variance. Among them, the first axis explains the majority of the variability in fish tolerance (48.7%), making it the most useful axis for modeling. TDG level (contribution rate = 59.7%, *p* = 0.002), species (contribution rate = 22.4%, *p* = 0.002), water temperature (contribution rate = 10.7%, *p* = 0.002) and fish weight (contribution rate = 3.5%, *p* = 0.014) significantly influenced the tolerance of fish in the upper Yangtze River, while the contribution rates of other factors were less than 1% and none reached statistical significance (Figure 3b). The result of hierarchical partitioning reveals that exposure time was the dominant factor influencing the mortality of fish in the upper Yangtze River in TDG-supersaturated water, with a contribution weight of 60.1% (*p* < 0.05). The contribution weights for TDG level, species and water temperature were 16.27%, 13.98% and 5.56%, respectively (*p* < 0.05). Water depth, fish length, fish weight and relative fatness contributed less than 3% and failed to reach statistical significance (Figure 3c). The result of random forest also identified exposure time, TDG level and water temperature as the top three key factors affecting fish mortality (Figure 3d). SHAP values from the random forest test increased with exposure time, TDG level and water temperature, indicating a positive impact on fish mortality. Conversely, relative fatness exerted a negative influence on fish mortality.

On the whole, exposure time, species, TDG level and water temperature were the top four factors affecting the mortality of fish in the upper Yangtze River based on the four statistical analyses, while the effects of relative fatness, body length, body weight and water depth were relatively weak and did not show significance. Using the Levenberg–Marquardt algorithm combined with a universal global optimization method, mathematical formulas with independent variables of exposure time, TDG level and water temperature for the mortality of rare fish and economic fish in the upper Yangtze River in TDG-supersaturated water are proposed as follows:

(1) $$P_{\mathrm{Mortality},\;M.A.}=(e^{t^{-0.7}-\ln(T^{2})-\ln\left(\frac{1}{\left(0.6+\mathrm{TDG}\right)}+T\right)}+T{)}^{\left(\mathrm{TDG}\times W-T-0.07\right)}$$

*P_Mortality, P.R._* = −7.7 + *TDG* × (0.17*W* − 0.5*T* + 0.42*t* + 11.6) − *W* × (3.32*T* + 0.23) + *T* × (0.02*T* − 0.02*t* − 0.13) (2)

(3) $$P_{\mathrm{Mortality}\;,S.P.}=\mathrm{EXP}\left\{\frac{T^{0.5}}{48.2}-\frac{4.5\times 10^{4}}{\left[1+800{\left(\mathrm{TDG}-1.23\right)}^{2}\right]\cdot \left[1+120{\left(t+0.43\right)}^{2}\right]}\right\}$$

*P_Mortality, E.F._* = (6.36*T*/*TDG*^−11.3^) × (1 + 0.26*t*) (4)

where *P_Mortality, S.P._*, *P_Mortality, M.A._*, *P_Mortality, P.R._* and *P_Mortality,__E.F._* represent the mortality of *S.P.*, *M.A.*, *P.R.* and economic fish, respectively, *t* (h) is exposure time, *TDG* (%) is the TDG level in water, and *T* (°C) is water temperature. These formulas demonstrated good predictive performance for the mortality of *S.P.*, *M.A.*, *P.R.* and economic fish within the 120–150% TDG range, achieving an *R*^2^ of 0.59, 0.81, 0.86 and 0.71, respectively.

### 3.3. Key Factors and Mathematical Formulas for Rare Fish Avoidance in TDG-Supersaturated Water

**Figure 4:**
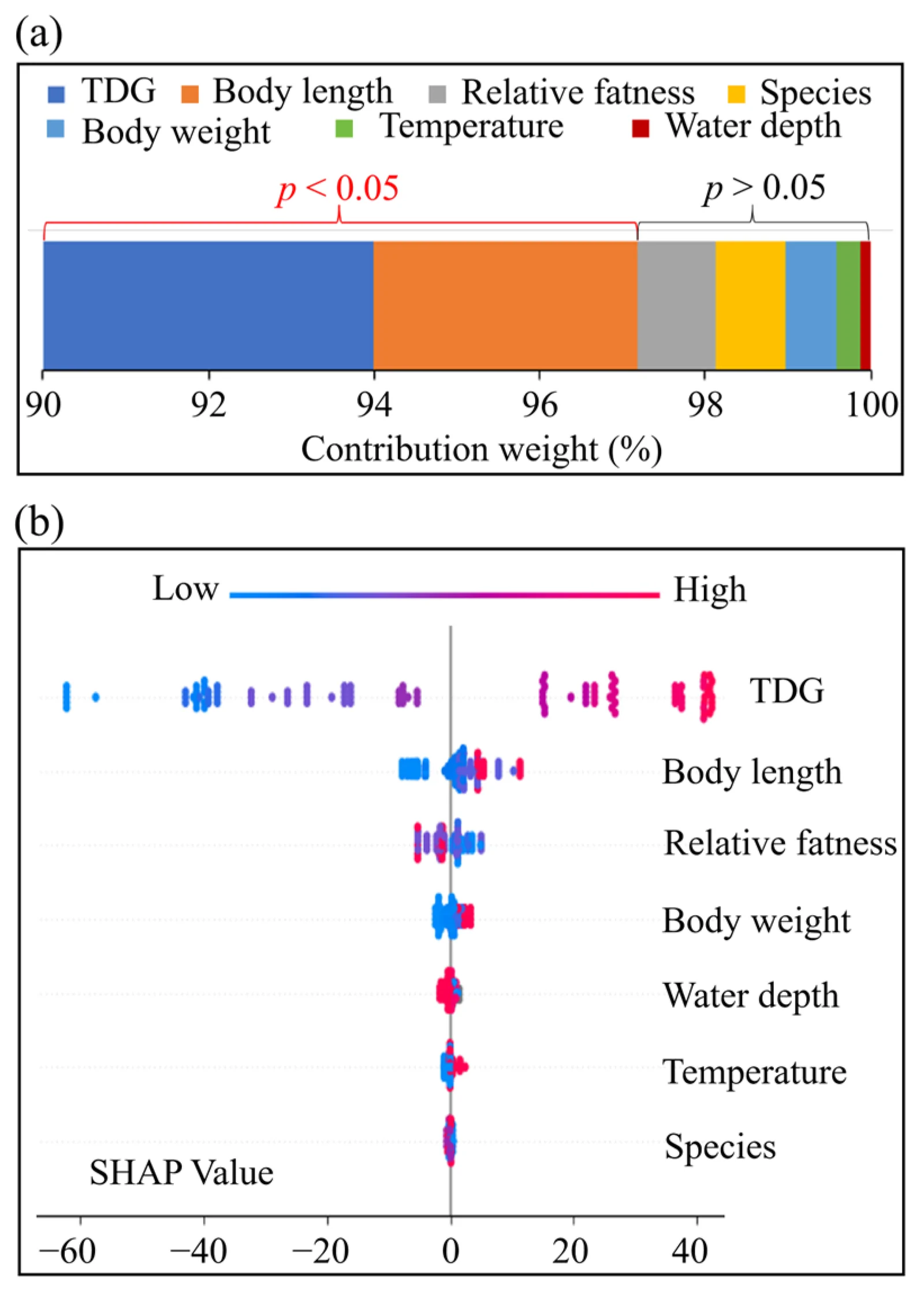
Factors affecting the avoidance of fish to TDG supersaturation by hierarchical partitioning via linear regression (**a**) and random forest (**b**).

Regarding fish avoidance to TDG supersaturation, the result of hierarchical partitioning revealed that TDG level significantly influenced horizontal avoidance rate (*p* < 0.05), exhibiting the highest contribution weight (94%) and thus playing a dominant role. Although fish length showed statistical significance (*p* < 0.05), its contribution weight to the horizontal avoidance rate was low at 3.71%. Water temperature, water depth, fish weight and relative fatness showed no significant effect on the horizontal avoidance rate (*p* > 0.05), with contribution weights below 1% (Figure 4a). The result of random forest revealed that the top three factors affecting the horizontal avoidance rate were TDG level, fish length and relative fatness (Figure 4b). TDG level and fish length exerted positive effects on horizontal avoidance rate, while relative fatness exerted a negative effect.

Generally speaking, TDG level was the most significant factor influencing the avoidance rate of rare fish species in the upper Yangtze River, with a contribution weight of 94% (*p* < 0.05) and ranking first in the random forest. Body length followed with a contribution weight of 3.71%, indicating that it also played an important role in fish avoidance behavior. The influence of other factors on the avoidance rate was relatively limited.

Similar to mortality prediction, this study identified TDG level and fish length, which significantly influenced fish avoidance, as independent variables, enabling us to propose a mathematical formula for the horizontal avoidance rate in TDG-supersaturated water as follows:

*A_h_* = 3.67 − 14.28*L*^−2^ − 10.68*e*^−*TDG*^ (5)

where *A_h_* (%) represents the horizontal avoidance rate of rare fish, and *L* (cm) is fish length. This model demonstrated good predictive performance for horizontal avoidance rates of rare fish within the 120–150% TDG range, achieving an *R*^2^ of 0.88.

## 4. Discussion

### 4.1. The Response Pattern of Fish Tolerance to TDG Supersaturation

The differences in fish tolerance to TDG supersaturation reflect the combined effects of species adaptability, biological characteristics and environmental factors [3,19]. This study confirmed that economic fish species had overall stronger tolerance than the rare fish species regardless of TDG level. This difference is the result of evolutionary divergence between the two groups in terms of geographic distribution and habitat adaptation strategies [29]. Rare fish in the upper Yangtze River have long been inhabiting environments with specific hydrological conditions, making them susceptible to environmental disturbances [30]. In contrast, economic fish species are not restricted to specific habitats, showing a broader distribution than rare fish, and can adapt to variable aquatic environments [31], endowing their physiological systems with greater buffering and regulatory capacity against bubble embolism caused by TDG supersaturation.

Exposure time, TDG level, and water temperature constitute the core factors influencing fish tolerance, which jointly regulate fish survival through different physiological pathways [32]. The positive correlation between exposure time or TDG level and mortality essentially reflects the cumulative physiological damage induced by TDG supersaturation [3]. As exposure time increases or TDG level rises, gases continuously enter the blood and tissues through fish gills, and then the partial pressure of dissolved gases in the blood and tissues increases accordingly. When the total gas pressure exceeds the sum of ambient hydrostatic and osmotic pressures, bubbles nucleate and grow directly within the vascular system and tissues. These bubbles cannot be readily resorbed, leading to vascular occlusion, tissue damage, and ultimately organ failure and mortality [33,34]. Water temperature as a key regulator of metabolism plays a dual role. Rising water temperature accelerates fish respiration and metabolic rates, which increases the uptake of dissolved gases and further exacerbates gas bubble disease [35]. In addition, fish enzyme activity and cell membrane permeability are subject to changes in water temperature [36]. These changes alter their ability to repair damage caused by gas bubbles. As a result, under the same TDG level, fish tolerance is always weaker in high-temperature environments.

This study found that factors such as body length, body weight, fatness and water depth did not significantly affect fish tolerance, which differs from the conclusions of some existing studies. The reasons for this discrepancy stem from the perspectives of experimental conditions and species characteristics. Regarding experimental conditions, the historical experimental data collected in this study featured a relatively narrow range of water depth gradients, failing to fully reflect the diluting effect of hydrostatic pressure on TDG supersaturation, and as a result, the compensatory effect of water depth on fish could not be quantified [11]. Regarding species characteristics, fish in this study were all at the juvenile stage, and differences in fish tolerance among juveniles were not reflected by their limited variation in body length and weight in this study [10,13]. This also suggests that the predictive formula of fish tolerance exhibits significant scale dependence. Future studies could incorporate broader gradients of body size, age, and environmental factors to more comprehensively elucidate the complex influences of multi-factor coupling.

Key factors have not been systematically identified in existing studies, while this study, for the first time, using multiple statistical methods, quantified the relative contributions of different factors to mortality and established a dynamic mortality prediction formula. The core significance of this study lies in providing a quantitative framework for prioritizing ecological risk management downstream of high dams. In practical engineering, controlling both the TDG level and duration of TDG may yield more significant reductions in fish mortality than simply lowering the TDG level alone.

### 4.2. The Response Pattern of Fish Avoidance to TDG Supersaturation

Fish avoidance behavior in response to TDG supersaturation encompasses the entire process of identifying, transmitting and responding to environmental signals [37]. When the TDG level was below 140%, these rare fish share similar sensory and behavioral response. These three rare fish species in the upper Yangtze River are all benthic or prefer the middle to lower layers of the water [20]. Their lateral line systems have long been shaped by the flow conditions of upstream rivers, making them similar in response to physical signals in the water [38]. This finding suggests that this convergent avoidance behavior reflects a common adaptive strategy among rare fish in the upper Yangtze River when coping with stress caused by high-dam discharges. Significant interspecific differences in fish avoidance were observed at a 140% TDG level or above, suggesting that there are potential differences in survival strategy when facing high lethal stress [14,20].

This study for the first time quantified contributions of different factors to fish avoidance, which confirmed that TDG level was the dominant factor, accounting for 94% of the effect, followed by body length at 3.71%, while the remaining factors had only a negligible impact. Fish mainly rely on their lateral line system to detect TDG supersaturation. Microbubbles in TDG-supersaturated water stimulate the sensory nerve endings along the lateral line and ultimately trigger avoidance behavior in fish, and species with smaller lateral line pores may experience more intense sensory stimulation [14,15,16]. In addition, acute exposure to TDG supersaturation induces significant physiological stress, which contributes to an overall stress state, and prompt avoidance without direct lateral line stimulation [39]. Hence, the overwhelming dominance of TDG level can be traced to its role as the trigger signal for avoidance behavior [40]. Higher TDG levels mean a stronger signal, which increases fish’s motivation to flee, leading to a marked rise in avoidance rates. The minor effect of body length on avoidance rates may stem from differences in sensory development. Larger juveniles typically possess a more developed lateral line system, making them more sensitive to bubble-related signals, which may explain their slightly higher avoidance rates [41,42].

Avoidance behavior is a proactive defense strategy that fish usually employ before experiencing lethal stress, and directly influences their chances of survival in TDG-supersaturated water [14,15]. Building on historical experimental data, this study established a quantitative predictive formula that linked avoidance rates to TDG levels and fish body length. This allowed us to achieve, for the first time, a quantitative prediction of avoidance behavior in rare fish species in the upper Yangtze River. This formula not only enables us to estimate the likelihood of fish avoidance under varying TDG levels, but also provides quantitative support for the selection and design of fish refuge areas downstream of high dams, thereby enhancing the effectiveness of conservation efforts.

Furthermore, there exists a clear transition between tolerance and avoidance. For rare fish species in the upper Yangtze River, the avoidance rate begins to exceed 50% at a TDG level of 130%, and this is exactly the point where their tolerance starts to drop sharply and mortality begins to rise. These findings reflect a survival strategy of warning–defense shaped by long-term evolution. Once environmental stress reaches the level that could cause physiological harm, fish instinctively take active steps to avoid it, thereby reducing their risk of death [40]. This finding suggests keeping TDG levels at a specific threshold at which minimum physiological damage to the fish and maximum fish avoidance efficiency are achieved.

### 4.3. Future Work

Based on numerous historical experimental data, this study revealed the interspecific difference in fish tolerance and avoidance in the upper Yangtze River in response to TDG supersaturation and established quantitative predictive formulas. However, due to limitations in experimental data and the scale of the study, certain limitations still remained, which point to directions for future research.

The data in this study were primarily derived from controlled laboratory experiments, where the experimental environment was idealized and homogeneous, differing significantly from the complex aquatic environment of natural rivers. In natural rivers, hydrodynamic conditions, including flow velocity and turbulent kinetic energy, are complex and variable, and there are interactions among multiple factors, while fish behaviors are also influenced by interspecific interactions and habitat heterogeneity [43]. Previous studies have shown that fish responded differently to TDG supersaturation in laboratory settings than they did in natural rivers [44]. In fact, the actual impact of TDG supersaturation on fish may be overestimated in indoor experiments [45]. A key reason for this is that fish in natural rivers have access to more refuge habitats, such as deep pools, tributaries, lotic and lentic areas, while complex hydrodynamic conditions help dilute TDG supersaturation and reduce environmental stress [46]. Future research should move beyond the constraints of laboratory experiments and place greater emphasis on in situ field studies. Taking into account the unique hydrodynamic and ecological characteristics of natural rivers, researchers can more accurately assess how fish respond to TDG supersaturation in real-world settings. This will help refine the predictive formulas built from lab experiments and make them more practically applicable.

From the perspective of practical engineering, the predictive formula developed in this study was mainly suited for fish species with similar ecological habits in the upper Yangtze River. However, rigorous validation through controlled experiments is an essential next step to translate these empirical models into practical management tools. Such validation efforts will not only strengthen the credibility of the current findings but also facilitate the application of these formulas in ecological risk assessments and fish conservation planning downstream of high dams. Since fish and hydrological conditions vary significantly across different rivers, future research can refer to the formulas established in this study and focus on cross-basin comparative studies to build a more universal predictive model which can be applied to a variety of river systems and fish species. At the same time, by combining hydraulic engineering operation strategies, the tolerance and avoidance behaviors of fish can be taken into account when designing spillway discharge plans. This would help establish a healthy river system that links engineering operations with ecological responses, supporting the balanced development of hydropower resources and the protection of aquatic biodiversity.

TDG supersaturation occurs not only downstream of dams, but also in natural processes such as waterfalls, rapids, sudden rises in water temperature, and oxygen production through photosynthesis in water bodies with high algal densities [11,47]. However, these natural events are typically less severe and no records of wild fish deaths associated with such events have been reported to date. This might be attributed to the behavioral adaptation of fish and the compensation of water depth. Research in this field is still limited and deserves further exploration in the future. Understanding both the natural and anthropogenic contexts of TDG supersaturation stress is essential for interpreting fish responses and developing effective conservation strategies.

## 5. Conclusions

This study revealed fish responses to TDG supersaturation, identified the key factors affecting fish tolerance and avoidance, and established mathematical formulas for fish tolerance and avoidance based on their primary drivers, from the perspective of historical experimental data in the upper Yangtze River. The following conclusions were reached:

At all TDG levels, economic species exhibited greater tolerance than rare fish. Significant interspecific differences in economic fish tolerance were observed with increasing TDG levels. *Aristichthys nobilis* demonstrated the strongest tolerance, followed by *Hypophthalmichthys molitrix*. For the rare fish, *Procypris rabaudi* showed the weakest tolerance. The avoidance ability of rare fish increased significantly with increasing TDG levels and reached 50% at 130% TDG. Significant interspecific differences in fish avoidance were observed at a 140% TDG level or above, and *Schizothorax prenanti* showed the weakest avoidance ability.

Exposure time, TDG level, species and water temperature were identified as the primary factors influencing fish mortality. Based on these, mortality prediction formulas for rare and economic fish were established, achieving an *R*^2^ over 0.59. In terms of avoidance, TDG level was the dominant factor, with 94% contribution, followed by body length. Accordingly, a prediction formula for fish avoidance rates was proposed with an *R*^2^ of 0.88.

## Figures and Tables

**Table 1:** Collected data on tolerance and avoidance characteristics of five endemic fish species in the upper Yangtze River under TDG supersaturation.

Experiments	Species	Parameters					
		TDG Level (%)	Sample Size	Body Length (cm)	Body Weight (g)	Water Temperature (°C)	Water Depth (cm)
Fish tolerance experiment under TDG supersaturation	*Schizothorax prenanti* [12,20,21,22]	120–150	1280	4.2–14.4	1.00–27.10	12–24	20–35
	*Procypris rabaudi* [20,23]	120–145	760	4.3–11.3	0.40–26.00	18–25	20–35
	*Myxocyprinus asiaticus* [20,24]	125–145	180	3.6–14.0	5.00–68.00	20	35
	*Hypophthalmichthys molitrix* [25]	130–150	100	4.8–13.8	2.68–17.33	25	35
	*Aristichthys nobilis* [26,27]	125–140	120	2.60–6.60	1.30–5.30	15	35
Fish avoidance experiment under TDG supersaturation	*Schizothorax prenanti* [20,21,22]	115–150	1760	6.0–14.0	4.0–50.0	21	18
	*Procypris rabaudi* [20]	105–150	1760	6.96–8.15	3.95–6.4	19–25	10–18
	*Myxocyprinus asiaticus* [20]	110–145	880	8.3	14.3	19	17

## Data Availability

The original contributions presented in this study are included in the Supplementary Material. Further inquiries can be directed to the corresponding author.

## Supplementary Materials

The following supporting information can be downloaded at https://www.mdpi.com/article/10.3390/ani16213423/s1: Table S1: The dataset on the tolerance and avoidance abilities of fish in the upper Yangtze River.

## Abbreviations

The following abbreviations are used in this manuscript:

TDG	Total dissolved gas
*S.P.*	*Schizothorax prenanti*
*M.A.*	*Myxocyprinus asiaticus*
*P.R.*	*Procypris rabaudi*
*H.M.*	*Hypophthalmichthys molitrix*
*A.R.*	*Aristichthys nobilis*
RDA	Redundancy analysis
*E.F.*	*Economic* fish
